# Supplementary material for: An artificial intelligence-based approach to identify volume status in patients with severe dengue using wearable PPG data
Source: PLOS Digit Health. 2025 Jul 18;4(7):e0000924. doi: 10.1371/journal.pdig.0000924 (PMC12273927; doi:10.1371/journal.pdig.0000924)
Supplement: S1 Table — (DOCX) [file pdig.0000924.s001.docx]

**S1 Table. Description of the HRV and waveform features.**

| Feature type | Feature | Description |
| --- | --- | --- |
| Heart rate variability | RMSSD | Root mean square of successive RR interval differences reflecting beat to beat variability, measure of vagal effects |
|  | SD1 | Poincare plot standard deviation perpendicular to the line of identity reflecting short term beat to beat variability |
|  | SD2 | Poincare plot standard deviation along the line of identity reflecting longer term variability |
|  | SDNN | Standard deviation NN intervals |
|  | Triangular index | Integral of the density of the RR interval histogram divided by its height reflecting overall variability |
|  | PNNI 50 | Percentage of successive NN intervals that differ by more than 50ms reflecting increased variability and correlated with parasympathetic activity |
| Waveform variability | SD duration diastolic decay | Standard deviation of the duration between the dicrotic notch and the beat end |
|  | SD area diastolic decay | Standard deviation of the area under the waveform between the dicrotic notch and the beat end |
|  | SD systolic width at 50% | Standard deviation beat width at 50% of the beat amplitude |
|  | SD duration LVET | Standard deviation of the duration of the left ventricular ejection time (from beat onset to the dicrotic notch) |
|  | SD area LVET | Standard deviation of the area under the waveform from beat onset to the dicrotic notch |
|  | SD duration crest | Standard deviation of the duration from the beat onset to the systolic peak |
|  | SD systolic amplitude | Standard deviation of the height of the systolic peak |
|  | SD systolic rise | Standard deviation of the slope of the rising edge of the systolic peak |
| Waveform beat durations | Mean duration LVET | Mean duration of the left ventricular ejection time (from beat onset to the dicrotic notch) |
|  | Mean duration crest | Mean duration from the beat onset to the systolic peak |
|  | Mean duration diastolic decay | Mean duration between the dicrotic notch and the beat end |
| Autonomic function | Mean HR | Mean heart rate |
|  | Mean NNI | Mean NN interval, negatively correlated with heart rate |
|  | SD2-SD1 ratio | Cardiac sympathetic index (CSI) which is an index of sympathetic activation [1] |
|  | LF | Low frequency power reflecting both sympathetic and parasympathetic activity |
|  | HF | High frequency power, also called the respiratory band and reflects primarily parasympathetic activity |
|  | LF-HF ratio | Reflects sympathetic dominance when high |

Reference

1. Balocchi R, Cantini F, Varanini M, Raimondi G, Legramante JM, Macerata A. Revisiting the potential of time-domain indexes in short-term HRV analysis. Biomed Tech Eng. 2006 Oct;51(4):190-3.
