# Supplementary material for: An artificial intelligence-based approach to identify volume status in patients with severe dengue using wearable PPG data
Source: PLOS Digit Health. 2025 Jul 18;4(7):e0000924. doi: 10.1371/journal.pdig.0000924 (PMC12273927; doi:10.1371/journal.pdig.0000924)
Supplement: S2 Table — (DOCX) [file pdig.0000924.s002.docx]

**S2 Table. Correlation table for the HRV and waveform features**


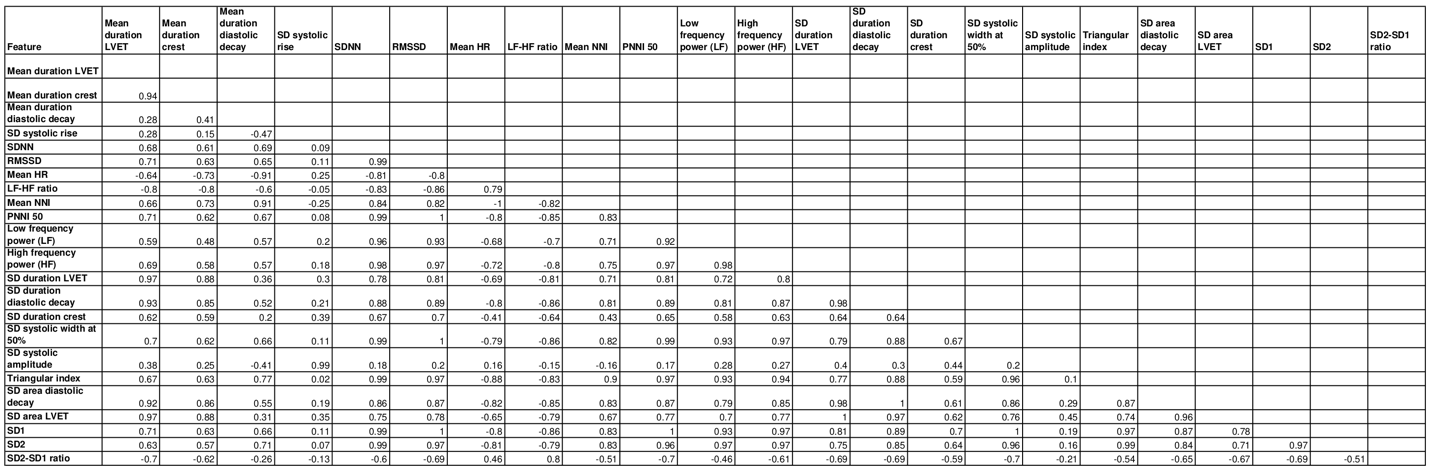


Note: Subject-level correlations are shown
