# Supplementary material for: An artificial intelligence-based approach to identify volume status in patients with severe dengue using wearable PPG data
Source: PLOS Digit Health. 2025 Jul 18;4(7):e0000924. doi: 10.1371/journal.pdig.0000924 (PMC12273927; doi:10.1371/journal.pdig.0000924)
Supplement: S3 Table — (DOCX) [file pdig.0000924.s003.docx]

**S3 Table. Baseline characteristics and outcomes.**

| **Characteristics** | **Patients enrolled in the study** | **Patients included in the analysis (study cohort)** | **Patients excluded from the analysis** |
| --- | --- | --- | --- |
|  | **Median (IQR) or Number (%) (n = 249)** | **Median (IQR) or Number (%) (n = 90)** | **Median (IQR) or Number (%) (n = 159)** |
| Age, years | 25.0 (16.0-33.0) | 19.0 (12.0-30.8) | 28.0 (19.0-34.0) |
| Children (<16 years) | 55 (22.1) | 35 (38.9) | 20 (12.6) |
| Adults (>=16 years) | 194 (77.9) | 55 (61.1) | 139 (87.4) |
| **Sex** |  |  |  |
| Male | 107 (43.0) | 36 (40.0) | 71 (44.7) |
| Female | 142 (57.0) | 54 (60.0) | 88 (55.3) |
| Body mass index, kg/m2 | 22.6 (19.8-26.0) | 21.7 (17.8-24.4) | 23.2 (21.4-26.6) |
| **Day of illness** |  |  |  |
| <3 | 10 (4.0) | 2 (2.2) | 8 (5.0) |
| 3 to 4 | 90 (36.1) | 28 (31.1) | 62 (39.0) |
| 5 to 6 | 141 (56.6) | 60 (66.7) | 81 (50.9) |
| >6 | 8 (3.2) | None | 8 (5.0) |
| Transferred from another hospital | 115 (46.2) | 40 (44.4) | 75 (47.2) |
| **Comorbidities** |  |  |  |
| Hypertension | 6 (2.4) | 1 (1.1) | 5 (3.1) |
| Diabetes | 12 (4.8) | 4 (4.4) | 8 (5.0) |
| **Physical exam signs and symptoms** |  |  |  |
| Bruising or petechiae | 189 (75.9) | 78 (86.7) | 111 (69.8) |
| Pleural effusion or rales | 75 (30.1) | 30 (33.3) | 45 (28.3) |
| Headache | 146 (58.6) | 51 (56.7) | 95 (59.7) |
| Vomiting | 124 (49.8) | 47 (52.2) | 77 (48.4) |
| Diarrhoea | 53 (21.3) | 23 (25.6) | 30 (18.9) |
| Abdominal pain | 145 (58.2) | 64 (71.1) | 81 (50.9) |
| Bleeding symptoms | 50 (20.1) | 16 (17.8) | 34 (21.4) |
| Respiratory symptoms | 50 (20.1) | 18 (20.0) | 32 (20.1) |
| **Vital signs on enrollment** |  |  |  |
| Temperature | 37.0 (37.0-37.5) |  | 37.0 (37.0-37.5) |
| Heart rate | 94.0 (83.0-105.0) | 95.5 (84.2-107.8) | 92.0 (83.0-103.0) |
| Systolic blood pressure | 110.0 (100.0-120.0) | 104.5 (100.0-110.0) | 110.0 (100.0-120.0) |
| Diastolic blood pressure | 70.0 (65.0-80.0) | 80.0 (70.0-80.0) | 70.0 (64.0-80.0) |
| Pulse pressure | 30.0 (30.0-40.0) | 30.0 (20.0-30.0) | 38.0 (30.0-47.5) |
| Respiratory rate | 22.0 (20.0-26.0) | 22.5 (20.0-26.0) | 22.0 (20.0-26.0) |
| **Laboratory values on enrollment** |  |  |  |
| White blood cells (k/uL) | 5.0 (3.2-6.5) | 4.6 (3.1-6.1) | 5.1 (3.3-6.7) |
| Hematocrit (%) | 45.0 (41.3-50.0) | 47.2 (43.6-51.1) | 44.5 (39.7-48.6) |
| Platelets (k/uL) | 22.0 (11.0-44.0) | 22.0 (12.2-33.8) | 22.0 (11.0-61.0) |
| AST (U/L) | 146.0 (77.0-339.0) | 145.0 (89.8-337.2) | 148.0 (70.5-344.0) |
| ALT (U/L) | 80.0 (38.0-206.0) | 76.5 (38.0-195.6) | 86.0 (38.5-206.5) |
| Creatinine (U/L) | 55.0 (50.0-73.0) | 53.5 (49.2-71.0) | 57.0 (50.0-75.0) |
| **Outcomes** |  |  |  |
| Patients with recurrent shock within 24 hours | 56 (22) | 29 (32) | 27(17) |
| Patients who received a colloid or crystalloid fluid within 24h | 178 (71) | 90 (100) | 84(53) |
