# Supplementary material for: An artificial intelligence-based approach to identify volume status in patients with severe dengue using wearable PPG data
Source: PLOS Digit Health. 2025 Jul 18;4(7):e0000924. doi: 10.1371/journal.pdig.0000924 (PMC12273927; doi:10.1371/journal.pdig.0000924)

S1 Fig. Example patient clinical courses showing how the “empty” and “full” states were defined in relation to clinical events. Reshock refers to recurrent shock. **A** An 11-year-old girl admitted to the pediatric ICU, **B** A 27-year-old woman transferred from an outside hospital, **C** A 17-year-old woman transferred from an outside hospital.


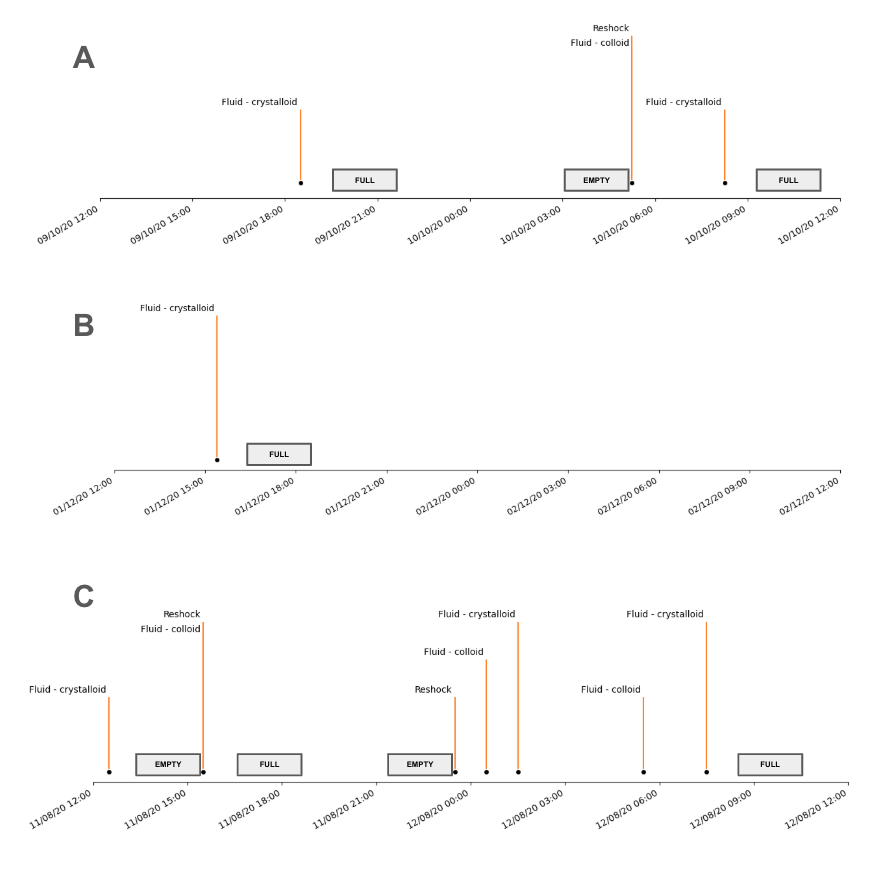

Supplement: S1 Fig — Reshock refers to recurrent shock. A An 11-year-old girl admitted to the pediatric ICU, B A 27-year-old woman transferred from an outside hospital, C A 17-year-old woman transferred from an outside hospital. (DOCX) [file pdig.0000924.s004.docx]
