# Supplementary figures and images for: An artificial intelligence-based approach to identify volume status in patients with severe dengue using wearable PPG data
Source: PLOS Digit Health. 2025 Jul 18;4(7):e0000924. doi: 10.1371/journal.pdig.0000924 (PMC12273927; doi:10.1371/journal.pdig.0000924)

**S2 Fig. Diagram of the fiducial points and waveform features.**


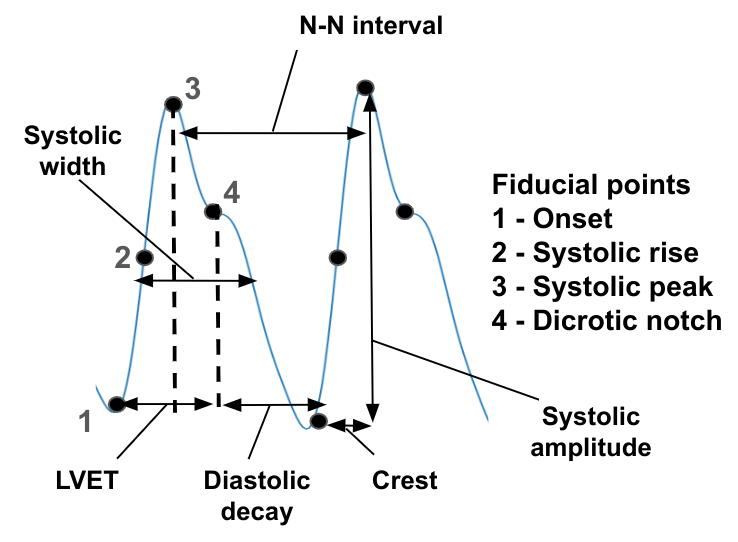

Supplement: S2 Fig — (DOCX) [file pdig.0000924.s005.docx]

**S3 Fig. ROC-AUC plots for the PCR and random forest.**


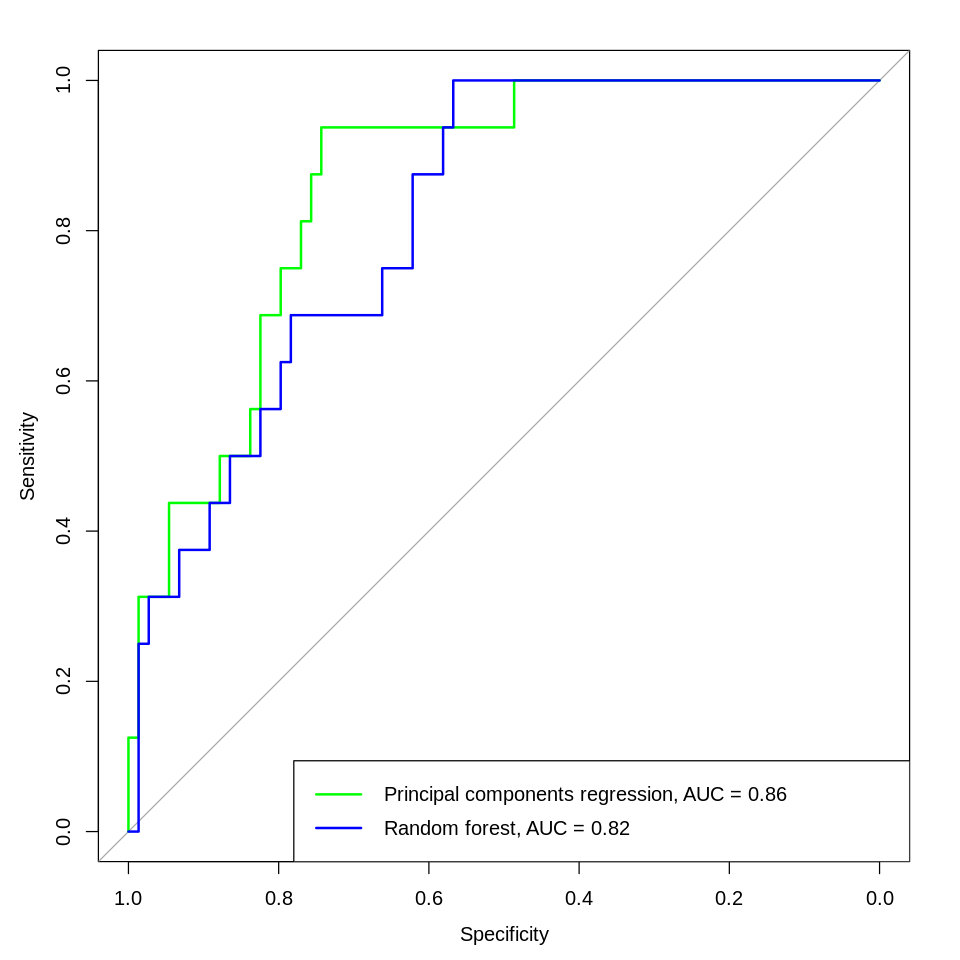

Supplement: S3 Fig — (DOCX) [file pdig.0000924.s006.docx]
