# Supplementary material for: An artificial intelligence-based approach to identify volume status in patients with severe dengue using wearable PPG data
Source: PLOS Digit Health. 2025 Jul 18;4(7):e0000924. doi: 10.1371/journal.pdig.0000924 (PMC12273927; doi:10.1371/journal.pdig.0000924)
Supplement: S4 Fig — (DOCX) [file pdig.0000924.s007.docx]

**S4 Fig. Confusion matrix and metrics for the vision transformer model.**


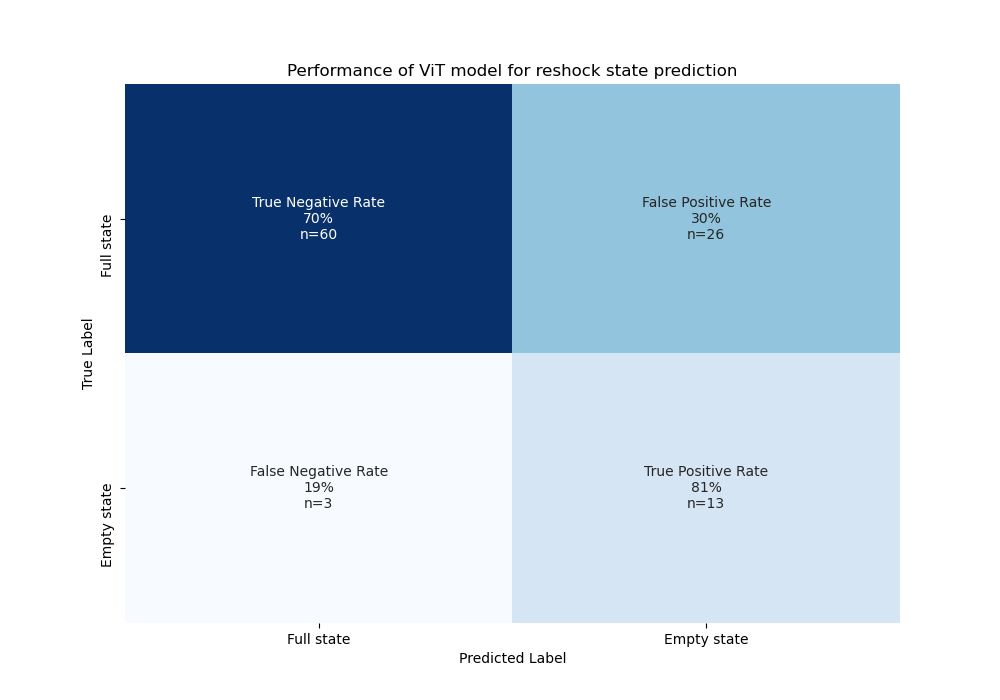


| **Metric** | **Interpretation** | **Value** |
| --- | --- | --- |
| Sensitivity | True positive rate or recall which is the proportion of correct predictions for the empty state | 0.81 |
| Specificity | True negative rate which is the proportion of correct predictions for the ‘full’ state | 0.70 |
| Precision | The proportion of ‘empty state’ predictions that are correct or how reliable the 'empty’ state predictions are | 0.33 |
| Accuracy | Proportion of total observations correctly classified | 0.72 |
| F1 score | The harmonic mean of precision and recall which is a better measure of accuracy for imbalanced datasets | 0.47 |
