# Supplementary material for: An artificial intelligence-based approach to identify volume status in patients with severe dengue using wearable PPG data
Source: PLOS Digit Health. 2025 Jul 18;4(7):e0000924. doi: 10.1371/journal.pdig.0000924 (PMC12273927; doi:10.1371/journal.pdig.0000924)
Supplement: Attachment — Submitted filename: Response To Reviewers.docx [file pdig.0000924.s009.docx]

# Manuscript PDIG-D-24-00435

**“An artificial intelligence-based approach to identify volume status in patients with severe dengue using wearable PPG data”**

We thank the editor and reviewers for their helpful comments and appreciate the time that you took to provide feedback. We have done our best to clarify our approach and address all of the comments and we believe that the manuscript is improved as a consequence. Please see below, for a point-by-point response to the reviewers’ comments. We address **each reviewer comment** in bold text, before highlighting alterations to the manuscript in plain text below. Page and line numbers refers to the updated manuscript (without tracked changes).

### Editor comments:

**Study Design and Clinical Framing**

**The binary classification approach needs substantial revision. As Reviewer 2 notes, the current framing of "before vs. after shock" has limited clinical utility.**

We thank the editor for highlighting this issue. To clarify, our study does not classify states “before” vs “after” a shock event. Our interest is to classify volume states. Toward this end, we designed our study to separate opposite volume states (“empty” vs. “full”) as a proof of concept. Regarding clinical utility, volume management is central to dengue shock management. Given the challenges around volume assessment during dengue shock (ie. lack of accurate/timely measurement and staffing limitations), a tool that improves volume assessment and potentially management would be very useful. Please see our response to Reviewer 2’s comments for further details regarding our approach and also updates to the manuscript to make this more clear.

**Please reframe the analysis to address more clinically relevant questions, such as predicting future shock events.**

As discussed above our goal is to classify two opposing volume states to demonstrate the proof-of-concept that PPG can be used to track volume changes rather than to predict shock. This is clinically relevant because volume assessment and volume management is central to dengue shock management.

**Include analysis of the excluded patients (n=159) who did not experience shock events, as these contain valuable physiological information for comparison.**

The type of analysis suggested by Reveiwer 2 to separate patients with dengue shock syndrome from those without has previously been done in our group (1). While it is of interest, this study focuses on heamodynamic volume status which we would argue is more clinically relevant to dengue management.

In addition, is not true that most of the patients in our cohort did not have shock events. Most of the patients (178/250 patients, 71%) in the study did have dengue shock syndrome meaning that they did have a shock event. However, half of these patients (88/178) were excluded from this study due to the lack of appropriate PPG data. This speaks to the challenge and complexity of conducting a pragmatic study in critically ill patients.

1. Karolcik S, Manginas V, Chanh HQ, Daniels J, Giang NT, Huyen VNT, et al. Towards a machine-learning assisted non-invasive classification of dengue severity using wearable PPG data: a prospective clinical study. EBioMedicine. 2024 Jun;104:105164.

**Statistical Analysis and Methodology**

**Address the sample size limitations and justify the use of advanced ML methods given the relatively small dataset.**

Machine learning and other advanced statistical methods are commonly used in datasets with as many or fewer observations than in our study and even in cases where there are many more features than observations. Good performance using a variety of ML methods have been demonstrated using real world datasets with as few as 10 observations (1). While we agree that our study would be strengthened if we had more patients, we believe that our methods support our conclusions. We discuss each of our methods below:

- For the logistic regression, we used principal components analysis (ML) to reduce the number of predictors to 4 from 23. The rule of thumb for logistic regression is about 10 observations per prediction (2). Therefore 90 patients is more than sufficient for this part of our analysis.
- For the random forest analysis, random forest is a robust method that can handle smaller datasets due to the use of bootstrap sampling and out-of-bag errors to generate performance metrics. Random forest is one of the best performing methods for the analysis of smaller datasets.
- For the computer vision model, there are 102 observations (i.e. patient-state combinations), however each observation represents up to 2 hours of PPG signal. As a result, multiple 20 second segments were sampled from each observation. Altogether we had greater than 6000 PPG segment images. This analysis could not have been done using conventional methods.

1. Fernández-Delgado M, Cernadas E, Barro S, Amorim D. Do We Need Hundreds of Classifiers to Solve Real World Classification Problems? Journal of Machine Learning Research. 2014;15:3133-3181.
2. Peduzzi P, Concato J, Kemper E, Holford TR, Feinstein AR. A simulation study of the number of events per variable in logistic regression analysis. J Clin Epidemiol. 1996;49(2):1373–1379.

**Clarify which definitions and choices were pre-specified (e.g., full/empty criteria, signal quality metrics).**

As we indicated in our manuscript the outcomes of “empty” and “full” were predefined based on previous work in our group suggesting that the period leading up to shock is the lowest volume state and the period following successful fluid initiation is the highest volume state.

For the principal components and random forest analyses, pulsewave features were selected based on availability (e.g. the features that could be computed by our statistical packages and measurements) and interpretability (based on our review of the literature). Waveform features relating to the amplitude of the waves were excluded a priori as discussed in our manuscript.

For signal quality we used device error codes, skewness, kurtosis and the presence for complete beat cycles. Signal segments were discarded when segments were associated with device error codes. Skewness and kurtosis values were not prespecified although suggested values in the literature were used as starting points. Our goal was to produce signal segments that were suitable for waveform morphology measures. Segments with incomplete beat cycles were excluded, again to enable waveform morphology measures.

**Claims and Interpretations**

**Revise statements about the algorithm's clinical utility. Provide evidence-based justification for claims about enhanced clinical care and timely interventions.**

Thank you for highlighting this issue. We have updated the manuscript throughout to emphasize that we are presenting an initial study to provide proof of concept rather than a tool for clinical implementation. While we are excited about the potential for the technology, we did not intend to suggest that our algorithm would enhance clinical care. We have changed some of the wording in our manuscript and removed some sentences to make this more clear. Please see our response to the Reviewers for further details.

### Reviewer #1 Comments:

**Summary and contributions**

**The manuscript explores an AI-based approach to monitor volume status in severe dengue patients using wearable photoplethysmography (PPG). It presents a prospective study of 250 patients, using machine learning techniques, such as principal components analysis (PCA), random forest, and a computer vision model. The study aims to classify "full" and "empty" hemodynamic states based on PPG data. Promising results include an 81% sensitivity for predicting the empty state using a vision transformer model and identification of sympathetic activation as a key indicator of volume depletion. This work underscores the potential of AI and wearable technology in dynamic and personalized fluid management in dengue shock syndrome (DSS).**

**The manuscript makes several important contributions:**

**1. It provides a novel application of PPG data and AI models in a clinical setting, focusing on volume status monitoring in DSS patients.**

**2. It highlights key physiologic markers of DSS, including sympathetic activity, and successfully applies both traditional machine learning (PCA and random forest) and deep learning approaches (vision transformers).**

**3. The study demonstrates the feasibility of using low-cost, non-invasive wearable technology for critical care in resource-constrained settings.**

We thank the reviewer for their kind words.

**Statistical Analysis Remarks**

**The following methods were applied:**

**1. Principal Components Analysis (PCA): PCA was used effectively to reduce dimensionality and highlight key physiologic factors (PC1 and PC4) linked to hemodynamic states.**

**2. Random Forest: This method identified important features (e.g., LF-HF ratio, mean heart rate) but had an out-of-bag error rate of 33%, limiting predictive reliability.**

**3. Computer Vision: The vision transformer model showed robust performance (81% sensitivity), though the F1 score (0.47) indicates challenges in handling imbalanced data. It will be beneficial to reference other vision models.**

Thank you for your comments. While model performance is of great interest, our primary goal was not to maximise performance but to provide proof of concept that PPG can be used for volume assessment in acute dengue shock syndrome. We also aimed to highlight the underlying factors/features that characterize the clinical states. Principal components regression and random forest are complementary methods for this type of analysis. We agree that our computer vision model struggled with the imbalanced data. However, we did try to address this issue by adjusting the threshold for prediction of the final state based on the average probabilities for all PPG segment images for each observation. As a result, we were able to improve sensitivity for the empty state, which is a clinical priority.

**Major**

**Statistical Methods: Although the statistical methods employed are appropriate for the study’s objectives, it will be beneficial to reference other ML/AI models that can be relevant for the analysis and their role in previous related studies.**

Thank you for this comment. We have updated our manuscript to include references (below) to other related studies in which other ML/vision models were deployed (page 12, lines 219-220).

1. Karolcik S, Manginas V, Chanh HQ, Daniels J, Giang NT, Huyen VNT, et al. Towards a machine-learning assisted non-invasive classification of dengue severity using wearable PPG data: a prospective clinical study. EBioMedicine. 2024 Jun;104:105164.
2. Ming DK, Daniels J, Chanh HQ, Karolcik S, Hernandez B, Manginas V, et al. Predicting deterioration in dengue using a low cost wearable for continuous clinical monitoring. NPJ Digit Med. 2024 Nov 2;7(1):306.

**Definition of Clinical States: The "full" and "empty" volume states are retrospectively defined and appear imprecise. A more granular classification or validation against additional clinical markers (e.g., echocardiography) could be used or at least referenced.**

We agree that the definitions for the volume states are less precise, particularly the “full” state. This was discussed as a limitation in our manuscript. Our definitions were informed by previous work done by our group using different non-invasive monitoring techniques such as compensatory reserve index (CRI) (1) and point-of-care echocardiography (unpublished data). These studies showed that markers of intravascular volume such as CRI and stroke volume index, decrease to minimal values as patients approach a shock event and they increase to maximal values about three hours after fluid is commenced.

As this is a pragmatic study, echocardiograms were not done for most of the patients, which reflects the reality of resource-limited settings where such assessments are often unavailable.

1. Trieu HT, Khanh LP, Ming DKY, Quang CH, Phan TQ, Van VCN, et al. The compensatory reserve index predicts recurrent shock in patients with severe dengue. BMC Med. 2022 Dec;20(1):109.

**Data Limitations: The study’s generalizability is limited by the homogeneous population (ethnicity and skin tone) and small sample size (90 patients). Addressing this limitation in future work should be emphasized more strongly. Can other methods be applied to strengthen the analysis.**

Thank you for your comment. We have updated the manuscript to emphasize the need to confirm our findings in larger patient cohorts with varied skin tones (page 21, lines 382-392)

With respect to the small size and methods, good performance using a variety of ML methods have been demonstrated using real world datasets with as few as 10 observations (1). We justify the our methods with regard to our sample size below:

- For the logistic regression, we used principal components analysis to reduce the number of predictors to 4 from 23. A commonly used rule of thumb for logistic regression is 5-10 observations per predictor.
- For the random forest analysis, random forest is a robust method that can handle smaller datasets due to the use of bootstrap sampling and out-of-bag errors to generate performance metrics. Random forest is one of the best performing methods for the analysis of smaller datasets.
- For the computer vision model, there are 102 observations (i.e. patient-state combinations), however each observation represents up to 2 hours of PPG signal. As a result, multiple 20 second segments were sampled from each observation. Altogether we had greater than 6000 PPG segment images analyses.

1. Fernández-Delgado M, Cernadas E, Barro S, Amorim D. Do We Need Hundreds of Classifiers to Solve Real World Classification Problems? Journal of Machine Learning Research. 2014;15:3133-3181.

**Feature Selection: While the exclusion of absolute amplitude features is justified, further explanation on how retained features (e.g., LF-HF ratio) avoid such biases would strengthen the methodology. Did the authors use/could use the random forest feature importance/SHAP/LIME/Other XAI methods?**

Thank you for this comment. We have updated the manuscript to include some discussion regarding the stability of the retained variables (page 21, lines 384-387). The retained variables including the LF-HF values are derived from the R-R interval (the time between the peaks of the individual beats) or from other time based measures. Much like heart rate which is calculated based on the number of peaks over time, the timing of peaks is not as likely to be different between different devices or skin tones.

**Data Quality and Preprocessing: The stringent quality control on PPG data led to reduced sample size. Exploring alternative preprocessing approaches to include more data could enhance model performance.**

We agree that less stringent quality pre-processing would allow for more of the data to be analyzed. Since our results were driven primarily by the pulse rate variability features, which have a lower quality requirement, rather than the waveform morphology features, which have a higher quality requirement, our study findings suggest that the analysis of lower quality signals using just the variability features may be adequate. This is something we plan to explore in future work.

### Reviewer #2 Comments:

**Summary**

**The findings are presented as proof of concept, which seems appropriate for the trial design and the modest accuracy.**

Response: Thank you for your comment.

**Major**

**1) I don’t follow the logic of a binary classifier that aims to separate physiological “state” before vs after a shock event. There is not a clinical circumstance in which I am asking, “is this patient's current state either before or after a shock event that was treated with fluids?”**

**The authors set this problem up as one of monitoring fluid status, which seems appropriate in so far as predicting future events (not asking am I before or after an intervention)**.

We thank the reviewer for the comment. However our study does not classify states “before” vs “after” a shock event. Our interest is in determining the utility of PPG data in classifying volume status rather than predicting future events. We designed our study to separate opposite volume states (“empty” vs. “full”) as an initial step. The ‘empty’ and ‘full’ states are not paired. As shown in Figure 2 of our manuscript only 12/90 patients had both “empty” and “full” events. Most patients (74/90) had only “full” events.

Based on our clinical experience and previous work done by our group (1), the 2 hour period leading up to a diagnosis of shock during the critical phase likely represents the lowest volume state. In contrast the 2 hour period about 1 hour after a “successful” fluid initiation event is likely to be closest to the highest volume state.

1. Trieu HT, Khanh LP, Ming DKY, Quang CH, Phan TQ, Van VCN, et al. The compensatory reserve index predicts recurrent shock in patients with severe dengue. BMC Med. 2022 Dec;20(1):109.

**Examples of how this can be framed include “PPG features can distinguish individuals who will vs will not, have a shock event in the next 24 hours”, or some other clinically relevant framing. As such, it is challenging to understand why the reader should be concerned with separability of before vs after shock events. At a minimum, the suggestion is to analyze all pre-shock event physiology to see if such a discriminator is possible - and if not, would severely limit the interpretation.**

As discussed above our goal is to distinguish two opposing volume states to demonstrate the proof-of-concept that PPG can be used to track volume changes. In our settings (low and middle income) it remains extremely challenging to estimate volume status in patients with dengue shock syndrome, which is essential to clinical management.

**2) I am concerned that the sample size here is inadequate to support anything other than descriptive statistics (rather than advanced ML).**

Machine learning and other advanced statistical methods are commonly used in datasets with as many or fewer observations than in our study and even in cases where there are many more features than observations. Good performance using a variety of ML methods have been demonstrated using real world datasets with as few as 10 observations (1). While we agree that our study would be strengthened if we had more patients, we believe that our methods support our conclusions. We discuss each of our methods below:

- For the logistic regression, we used principal components analysis (ML) to reduce the number of predictors to 4 from 23. The rule of thumb for logistic regression is about 10 observations per prediction (2). Therefore 90 patients is more than sufficient for this part of our analysis.
- For the random forest analysis, random forest is a robust method that can handle smaller datasets due to the use of bootstrap sampling and out-of-bag errors to generate performance metrics. Random forest is one of the best performing methods for the analysis of smaller datasets.
- For the computer vision model, there are 102 observations (i.e. patient-state combinations), however each observation represents up to 2 hours of PPG signal. As a result, multiple 20 second segments were sampled from each observation. Altogether we had greater than 6000 PPG segment images. This analysis could not have been done using conventional methods.

1. Fernández-Delgado M, Cernadas E, Barro S, Amorim D. Do We Need Hundreds of Classifiers to Solve Real World Classification Problems? Journal of Machine Learning Research. 2014;15:3133-3181.
2. Peduzzi P, Concato J, Kemper E, Holford TR, Feinstein AR. A simulation study of the number of events per variable in logistic regression analysis. J Clin Epidemiol. 1996;49(2):1373–1379.

**3) The authors seem to mis-construe the inferences based on their framing and on the “sensitivity”, by stating in the discussion that 81% sensitivity means the method has utility in predicting future shock events. As I understand the framing, the algorithm does predict future shock events. Even if it did (per suggestion in comment #1 for new analysis, the text about sensitivity seems to be conflating PPV with sensitivity. By their own dichotomous report, where specificity is lower value (even though the post-shock full stats is the not the “negative” of a pre-shock empty state), a test with higher sensitivity than specificity would tend to have better rule-out, than rule-in, performance (although the prior is the main determinant obviously).**

Again our goal is to separate high from low volume states rather than to predict shock. We have updated our manuscript throughout to make this more clear. The sensitivity metric refers to the true positive rate which is an output of the computer vision model as shown in the confusion matrix in Supplementary Figure 3.

**4) The authors speculate that PPG might somehow be “more informative” than clinical assessment of volume - this seems like quite a stretch - no attempt was made to compare the PPG results with clinical predictions here. At best, the authors might speculate that some PPG features could be used as adjunct to clinical assessment (rather than jump all the way to PPG being more informative - no comparison was made of, say, PPG vs clinical assessment).**

Thank you for this comment. We did not intend to suggest that PPG is “more informative” compared to clinical assessment and our study would not support that conclusion. We have rewritten the relevant sentence in our manuscript to clarify (page 19, lines 341-343).

**5) I don’t understand the claim that PPG somehow frees up nursing resources due to taking vitals less frequently - again, seems like a large stretch in many respects - if nurses are even taking vital signs (vs some other support staff plus telemetry if the person is sick enough to worry about shock), this feels like a minority of a shift’s burden, and given the core necessity of vitals in critical care, it seems implausible vitals will ever be fore-gone in favor of some black box algorithm?**

At our center (The Hospital for Tropical Diseases in Ho Chi Minh City), patients admitted with dengue shock syndrome have vitals taken every 15 to 30 mins initially and then hourly for at least for 24-48 hours. Vital signs are measured manually by nurses. In addition, urine output and haematocrit are assessed hourly. There is very limited access to telemetry and no additional support staff for patient monitoring. During peak dengue season, when patient numbers can surge by 200–300%, the work of monitoring patients can be overwhelming. A single nurse may be responsible for 3-4 shock patients. We are not suggesting that PPG monitoring will replace clinical assessments, however a device that reduces the burden of frequent manual monitoring would be beneficial in our center and likely in other resource-constrained settings.

**6) The discussion speculation is unjustified: there is no evidence in the report that the algorithm is accurate enough to enhance clinical care, or to make fluid interventions more “timely”. Again, the prediction was not set up in this paper, as far as I understand, as one of “will this patient have a shock event or not”. Instead it was set up as “is this patient before or after a shock event followed by a successful fluid intervention”. Even if the authors do this, they would presumably need to march through clinical examples taking FP and FN rates and risks into account to justify questions like "how accurate would this need to be for clinical implementation?"**

Our goal is to provide proof of concept that PPG can be used to monitor volume status during dengue shock syndrome. To this end we examined the most extreme volume states during this period and showed that we could separate these states based on the PPG. In addition we also highlighted a few underlying physiologic factors/features. Given the importance of volume management in severe dengue we see significant potential for the technology. However our algorithm is not meant for clinical implementation.

**7) Most patients in this cohort (n=159 it seems) were excluded, a large portion of which seem did not have a shock event - but these subjects presumably carry important physiological information and should be analyzed. It seems for example very important to compare the PPG features of those excluded (no shock) individuals against those who did have a shock event (the “empty” cohort). This would greatly strengthen the story, and align with the proposed importance (a future clinical prediction tool to aid in clinical care).**

The type of analysis to separate patients with dengue shock syndrome from those without has previously been done in our group (1). While it is of interest, this study focuses on heamodynamic volume status which we would argue is more clinically relevant.

It is not true that most of the patients in our cohort did not have a shock event. In fact, most of the patients (178/250 patients, 71%) in our study did have dengue shock syndrome meaning that they did have a shock event. Half of these patients (88/178) were excluded from this study due to the lack of appropriate PPG data. This speaks to the challenge and complexity of conducting a pragmatic study in critically ill patients.

1. Karolcik S, Manginas V, Chanh HQ, Daniels J, Giang NT, Huyen VNT, et al. Towards a machine-learning assisted non-invasive classification of dengue severity using wearable PPG data: a prospective clinical study. EBioMedicine. 2024 Jun;104:105164.

**8) Related to the above, some additional clarifications will help the labeling. For example, “Full” state is not just fluid repleted, but also clinically improved. So, it seems, we have patients without shock events (presumably none got fluids?), and then we have those with shock events (presumably all got fluids then?), and of those, some that recur or are unstable/poorly responsive. Seems it would be very helpful to chart out the distribution of these possibilities, which will also set up more obviously when and how PPG based fluid status might be useful in clinical flow.**

We confirm that the clinical study enrolled patients without shock who were not treated with fluids. These are patients who were admitted with dengue with warning signs but who ultimately did not develop shock. We also confirm that any patient with shock at any time during their illness did receive fluid. However, not all shock and/or fluid initiation events occurred during the 24 hour period following enrollment. For example some patients were transferred from an outside hospital, in which case they would have had shock or fluid initiation prior to study enrollment. We agree that a follow up study examining the PPG signal among different patient subgroups in relation to their clinical course is of interest however that was not the aim for this study.

**9) Please indicate which definitions and choices were pre-specified (full and empty criteria? Signal quality metrics? etc).**

As we indicated in our manuscript, the outcomes of “empty” and “full” were predefined based on previous work in our group suggesting that the period leading up to shock is the lowest volume state and the period following successful fluid initiation is the highest volume state.

For the principal components and random forest analyses, pulsewave features were selected based on availability (e.g. the features that could be computed by our statistical packages and measurements) and interpretability (based on our review of the literature). Waveform features relating to the amplitude of the waves were excluded a priori as discussed in our manuscript.

For signal quality we used device error codes, skewness, kurtosis and the presence for complete beat cycles. Signal segments were discarded when segments were associated with device error codes. Skewness and kurtosis values were not prespecified although suggested values in the literature were used as starting points. Our goal was to produce signal segments that were suitable for waveform morphology measures. Segments with incomplete beat cycles were excluded, again to enable waveform morphology measures.

**Minor**

**1) I assume the LF-HF ratio was computed from segments longer than 20 seconds. This should be explained. For example in the supplemental table, indicate for each metric the time window used to compute the feature.**

All segment features, including the LF-HF ratio were computed using the 20 second segments. This was done using the HRV package in python.

**2) Acronyms should be explained in all cases (e.g. loading plots, feature abbreviations), in the main text (not just in the supplement).**

Thank you for this comment, we have updated the manuscript (page 16, lines 273-277) and added figure captions to the loadings and feature importance plots.

**3) The “discarded” rules are not well articulated - at a minimum, the authors should indicate the percent of PPG signals not meeting the “discard” criteria.**

We have elaborated on this in the updated manuscript (page 9, lines 158-164).

**4) Suggest indicating in the correlation matrix whether the individual points are being correlated or whether a mean was taken per subject and the correlation is done at the subject level (the latter of which will often have much higher R values).**

The correlation was done at the subject/patient level. The mean of each feature was calculated, from the individual segments for each patient and the correlation was determined between these means. We have updated the manuscript to make this clearer (page 15, lines 260-261) and added a note to the correlation table in the Appendix.

**5) I do not understand the authors claiming sympathetic activity in shock is a novel finding - seems like a classic mechanism?**

We agree that one would expect heightened sympathetic activity as dengue patients become hypovolemic and progress to shock. However, the finding of a “digital marker” of sympathetic dominance from the principal components analysis is novel and has not been described in dengue shock. By identifying and naming this factor, it then becomes possible to measure and follow it. We have revised some of the wording in our manuscript to make this clearer (page 19, lines 330-332).

**6) This bold claim would seem to require some citation(s): “PPG monitoring provides a more dynamic and complete picture of the haemodynamic volume state compared to other methods like echocardiography.” It is insufficient to simply remark that your team has anecdotal observations on this.**

Thank you for this comment. We have removed this sentence from our manuscript.

**7) I am unclear on the timing of the PPG vs the shock events. Was the empty state really “any” time before shock?**

As noted in the methods, the empty state was defined as being the 2-hour period leading up to the clinical diagnosis of shock, so the empty state was not “any” time before shock.

**Suggest making a figure or some description of these temporal distributions. For example, you could have 90 rows, where X axis is time in days from enrollment, with the time of the PPG indicated, and the time of shock indicated, maybe color shading of when you say the empty vs full periods are.**

Response: Thank you for your suggestion. Patients undergo PPG monitoring and recording of clinical events (shock and fluid initiation) for only 24 hours after enrollment. We have added a figure showing examples of patients’ clinical course relative to the predefined volume states in the Appendix (page 32, Supplementary Figure 1).

**8) I don’t understand Suppl. Fig 1. It appears the authors have simply ordered the p(state) for empty or full - what is the meaning of such a plot?**

This plot is just a simple visualization of the performance of the principal components regression model, showing that the empty state patients tend to have a higher probability of being in the empty state. However, we appreciate that it is a non-standard plot which may cause confusion and have removed it in our revised manuscript.

**Reviewer #3 Comments:**

**Summary**

**The study's methodology is robust and thoughtfully designed, demonstrating a strong potential to address existing challenges in fluid management for DSS. The development of a DSS-specific algorithm is a key strength, as it could streamline clinical workflows and reduce the burden on paramedical staff. Additionally, the study highlights its limitations, such as the need for larger-scale validation or potential variability in PPG signal quality, which strengthens the credibility of its findings. Overall, this is a well-conducted study that makes a meaningful contribution to the field. Its findings pave the way for future research, particularly in refining the algorithm and exploring its implementation in diverse clinical settings.**

Thank you for your positive comments.

**Major**

**1. The concepts of “empty state” and “full state” are central to this study. Initially, these terms give the impression of being interdependent and occurring in pairs during an "event." However, it becomes clear later in the manuscript that they are independent variables and can be treated separately. To reduce potential confusion, it would be helpful to clarify early in the manuscript that these states do not necessarily occur in pairs for a shock event. Additionally, these concepts appear to be novel and are uniquely defined or inferred based on adjacent clinical events. Highlighting their novelty upfront would provide readers with a clearer understanding of their importance and contribution to the study's framework.**

Thank you for this excellent suggestion. We have updated the manuscript to clarify that they do not necessarily occur in pairs and that they are novel concepts (page 8, lines 144-146). We have also added a new figure to the appendix showing examples of patients' clinical courses and how the volumes states were defined (page 32, Supplementary Figure 1).

**2. The decision to exclude absolute amplitude values, such as the height of the systolic peak or the area under the beat, due to the homogeneity of the study population (single skin color) is a thoughtful attempt to address potential biases. However, it would be valuable to discuss whether this exclusion might introduce any limitations. For instance, while using variation measures (e.g., the standard deviation of systolic peak heights) offers consistency, will it reduce sensitivity to any critical variables? Per research, we know that a decrease in pulse amplitude often serves as an early indicator of impaired circulation or shock. Could this focus on variation measures lead to the algorithm overlooking some of these early, critical signs? Additionally, will this approach risk overfitting to the specific characteristics of the study population or experimental conditions? Discussing these potential trade-offs would strengthen the manuscript and provide a more comprehensive understanding of the methodology’s implications.**

Again thank you for this thoughtful suggestion. We agree that the amplitude of the waveform reflects intravascular volume. In sensitivity analyses we did include the amplitude features and this resulted in no significant difference to our results overall. Ultimately we removed features because we felt we could not justify keeping them and in order to improve the robustness of our models for future use in other populations. However, we agree that if we could correct for the variation in amplitude that occurs due to skin tone and/or device positioning/fit, that this may lead to more accurate results. With respect to the focus on variation measures, we agree that the models may be less generalizable to other races/ethnicities since pulse rate variability itself is different between different races/ethnicities. We have updated the manuscript to discuss these considerations (page 21, lines 383-392).

**3. Given the limited number of studies exploring the use of CRI to predict recurrent shock in DSS patients, it would be valuable to include an explanation of how this new algorithm sets itself apart from the features used to calculate CRI.**

Unfortunately the CRI is a proprietary measurement (1) so it is not possible to compare features with those from our models. One of the factors that motivated our study, and our interest in PPG for dengue monitoring was a previous study by our group (2) describing CRI changes during dengue shock syndrome showing that the CRI correlated with clinical volume states. However, since the CRI is proprietary and costly, it can not be widely implemented at our hospital.

1. Moulton, Steven L. MD; Mulligan, Jane PhD; Grudic, Greg Z. PhD; Convertino, Victor A. PhD. Running on empty? The compensatory reserve index. Journal of Trauma and Acute Care Surgery 75(6):p 1053-1059, December 2013. | DOI: 10.1097/TA.0b013e3182aa811a
2. Trieu HT, Khanh LP, Ming DKY, Quang CH, Phan TQ, Van VCN, et al. The compensatory reserve index predicts recurrent shock in patients with severe dengue. BMC Med. 2022 Dec;20(1):109.

**Minor**

**1. The low sample size for the "empty" population in the training set seems like it might be a limitation. It would be beneficial to expand on how this might impact the generalizability of the findings? It would be great to understand if there are ways this was addressed or if it opens up opportunities for future work.**

We agree that imbalanced data is a challenge, particularly for the computer vision model. All procedures to mitigate this were described in the manuscript. For the principal components regression, no procedures were applied to address this. Despite this the model performed well with an AUC of 0.86. For the random forest we undersampled the majority state (i.e. the full state) to balance the labels for each tree and specified a large number of trees (ntree = 1000) to ensure that the model had adequate exposure to the full state data. We achieved good performance with an AUC of 0.82. The ROC-AUC plots for both these analyses are shown in the Supplementary Figure 2 in the Appendix. For the computer vision model we considered many techniques including undersampling the “full” state and augmenting the “empty” state. Ultimately, in consultation with a computer scientist (James Anibal who is the 3rd author), we adjusted the threshold for prediction of the final state based on the average probabilities for all PPG segment images for each observation so as to improve sensitivity for the “empty” state, which is a clinical priority.

**2. The term "explainable" dengue-specific algorithm is mentioned in the introduction, and it would be helpful to clarify what "explainable" means in this context. Does it suggest that the algorithm will provide doctors with insights into why a particular status is determined for a subject? If so, it would be interesting to learn more about how this "explanation" is incorporated into the algorithm’s design.**

The term “explainable” was meant to contrast our algorithms with the CRI algorithm which is proprietary, however we appreciate the confusion with this word and have updated the terminology in our manuscript (page 6, lines 97-100).

**3. It would be nice to include details about the envisioned practical application for this algorithm. For instance, could it be integrated into bedside tablets or other monitoring systems? A brief description of how it might be used in clinical settings would provide helpful context for its potential impact.**

Response: Thank you for your comment. Our study used wrist worn devices connected to bedside tablets via bluetooth. Data were then manually transferred to hospital servers daily. Since the study was completed, a hospital wide network has been created for the real-time transfer of data from the tablet to hospital servers via wifi. If data can be transferred to servers in a timely way, predictions can be generated to guide fluid management. However, since our algorithms separate only extreme volume states, further work is needed before clinical implementation. Ideally we would be able to track volume status at several levels or along some continuous scale as with the CRI.

**Overall, this study presents a promising approach to fluid management in DSS patients, and the development of a DSS-specific algorithm is an exciting contribution to the field. Addressing the points mentioned above would further strengthen the manuscript and provide additional clarity for readers.**

Response: Thank you for your kind comments!
