# Supplementary material for: An artificial intelligence-based approach to identify volume status in patients with severe dengue using wearable PPG data
Source: PLOS Digit Health. 2025 Jul 18;4(7):e0000924. doi: 10.1371/journal.pdig.0000924 (PMC12273927; doi:10.1371/journal.pdig.0000924)
Supplement: Attachment — Submitted filename: Response To Reviewers Apr9.docx [file pdig.0000924.s010.docx]

# Manuscript PDIG-D-24-00435

**“An artificial intelligence-based approach to identify volume status in patients with severe dengue using wearable PPG data”**

We thank you for your comments and appreciate the time that you took to provide feedback. Please see below, for our response (in plain text) to each comment (in bold text). Page and line numbers refers to the updated manuscript (without tracked changes).

### Editor comments:

**The changes look appropriate. Please address the reviewer comments regarding binary classification and clarifying a few additional details.**

Thank you, please see our response below.

### Reviewer #2 Comments:

**1) regarding the binary classification, I am still a bit confused. The authors state that “empty” is always before resuscitation, and “full” is always after a shock event (I think we all agree here). Yet they also say in their response, quote: “our study does not classify states “before” vs “after” a shock event.” These seem incompatible statements. The language feels relevant to my comment #2.**

To clarify while “empty” is always before a shock event, “full” is always after a fluid initiation event but NOT always after a shock event. That is while a shock event is typically followed by a fluid initiation event, a fluid initiation event is NOT always preceded by a shock event. This is why we stated that “our study does not classify states before vs after a shock event”.

We revised our manuscript to be clear on how the “empty” and “full” states were defined (pages 6-7, lines 134-148) because they are novel concepts which are central to our study. We also added a new figure to the appendix showing examples of patients' clinical courses and how the volumes states were defined relative to the clinical events (page 32, Supplementary Figure 1). As shown in the figure, many fluid initiation events and “full” events are not preceded by a shock event. Fluid management in severe Dengue is at the discretion of the treating physician and fluids can be initiated in the absence of a preceding shock event. The goal is to maintain perfusion in the setting of ongoing plasma leakage so patients may have fluid initiated if they are deemed to be at risk.

**2) It feels like the most relevant comparison for your goals seems to be shock vs no shock. If the authors are unwilling to analyze the features from the no-shock group (n=71, it seems, see next comment), it feels like it should be called out directly in the discussion/limitations, that this classifier framing for proof-of-concept is distinct in nature from what is desired in the future: that it will need to run with inputs from pre-shock data, to bridge the gap from this work to a future clinical implementation (prediction) task. As written, especially with the emphasis on sensitivity of 81 (without even calling out the specificity - which by the way is listed as 67 in abstract, but 70 in the paper, and needs to be reconciled please), makes it seem as though this comparison is actually the desired one for future implementation.**

Thank you for pointing out the discrepancy in the specificity value. We have corrected this in the revised manuscript (page 2, line 47). In respect to the analysis of the PPG data from the patients who were admitted with dengue but who ultimately did not develop shock or require IV fluids (without dengue shock syndrome), we appreciate the suggestion which we will consider for future work.

**3) My comment #7 did not say most subjects did not have shock. It said most were excluded (159, per your diagram, is more than have of consented total), and of those, “a large portion” did not have shock (n=71). This is pertaining to comment #2, which refers to the question, why not analyze those 71 for the same features, to see if those who did go into shock are different than those who didn’t? Seems highly relevant, and would enormously improve the impact of this work.**

Again, we appreciate the suggestion to study patients without dengue shock syndrome which we will consider for future work. In this study we wanted to focus on volume status among patients with dengue shock syndrome who are actively being treated. Our approach is motivated by previous work done by our group using the compensatory reserve index (1) and point-of-care echocardiography (unpublished data) which suggested that intravascular volumes are lowest leading up to a shock event and they are highest about three hours after a “successful” fluid initiation event.

1. Trieu HT, Khanh LP, Ming DKY, Quang CH, Phan TQ, Van VCN, et al. The compensatory reserve index predicts recurrent shock in patients with severe dengue. BMC Med. 2022 Dec;20(1):109.

**4) regarding the sample size, the authors have quoted a paper (Do We Need Hundreds of Classifiers to Solve Real World Classification Problems?) to indicate small sample sizes are acceptable. However, this is not the conclusion of that paper, to my read, which defined “maximum accuracy” as the top accuracy across many classifiers applied to a single data set. This is a very different question from what is at stake here: is the sample size sufficient to make inferences about the problem at hand. This is not simply a statistical question applied indiscriminately (although it is often framed as such), it depends on the complexity of the input signals or features, the uncertainty in the labels, among many other factors. My suggestion is to call out the small sample size in the limitations.**

We have revised the discussion to include small sample size as a limitation (page 22 , lines 413-419).

The paper “Do We Need Hundreds of Classifiers to Solve Real World Classification Problems?” (1) describes the performance (i.e. accuracy) of a variety of machine learning methods on a collection of 121 real-world datasets. Two aims of the paper are to “to rank each classifier and family according to its accuracy” and to “to evaluate the classifier behavior varying the data set properties (complexity, #patterns, #classes and #inputs)”. The number of observations (which is referred to as #patterns) in these datasets range from 10 to >130,000. Table 1 from this paper shows the number of observations for each dataset under the heading “#pat”. As shown, many of the datasets have 100 or fewer observations. We cited this study to show that machine learning can be used to analyze smaller datasets, with good results. However, we agree that the performance of an algorithm depends on the characteristics of the data, in particular the signal:noise ratio, and that larger sample sizes are needed when the signal:noise ratio is not high.

1. Fernández-Delgado M, Cernadas E, Barro S, Amorim D. Do We Need Hundreds of Classifiers to Solve Real World Classification Problems? Journal of Machine Learning Research. 2014;15:3133-3181.

**5) the authors responded to my question that the empty state was not “any” 2 hours period - but their methods continue to read, and I quote: “An “empty” state was any two-hour period before a shock event.” (Page 6, line 137, in the marked version). Please amend the methods to align with your response to my question.**

Thank you for this comment. We have removed the word “any” in the revised manuscript for improved clarity (page 6 line 134 and page 7 line 138).

### Reviewer #3:

**Thank you for the updated manuscript. The Authors have made the necessary updates to their manuscript based on the my review comments. I believe that this study greatly adds to the existing body of research in DSS fluid management domain.**

Thank you for your comments and encouraging feedback!
